# Pseudomyopia with paradoxical accommodation: a case report

**DOI:** 10.1186/s12886-021-01907-5

**Published:** 2021-03-31

**Authors:** In Ki Park, Young Kee Park, Jae-Ho Shin, Yeoun Sook Chun

**Affiliations:** 1 Department of Ophthalmology, Kyung Hee University College of Medicine, Kyung Hee University Hospital, Seoul, Korea; 2 YK Eye Clinic, Seoul, Korea; 3 Department of Ophthalmology, Kyung Hee University College of Medicine, Kyung Hee University Hospital at Gangdong, Seoul, Korea; 4 Department of Ophthalmology, Chung-Ang University College of Medicine, Chung-Ang University Hospital, 102 Heukseok-ro, Dongjak-gu, 06973 Seoul, Korea

**Keywords:** Accommodation, Case report, Ciliary muscle, Paradoxical, Pseudomyopia

## Abstract

**Background:**

Pseudomyopia is caused by increased refractive power by ciliary muscle spasm. Most patients cannot overcome pseudomyopia spontaneously; therefore, treatment of pseudomyopia is fastidious and needs a multidisciplinary approach. We report a case of unusual pseudomyopia with paradoxical accommodation, straining eyes to induce emmetropia at far distance and relaxing eyes to focus at near objects, contrary to physiological accommodation.

**Case presentation:**

A 33-year-old woman experienced intermittent distant vision discomfort. This occurred at least a few hundred times daily. She could see near objects clearly; however, distant objects could be seen clearly only when she strained her eyes. Uncorrected distance visual acuity was 20/20 and manifest refraction (MR) in both eyes in the relaxed state was approximately − 2.5 D. MR changed to approximately − 0.5 D when she grimaced and strained her eyes when attempting to focus on distant letters. Her response was contrary to the physiological accommodative response. Cycloplegic refraction was approximately 0.0 D. Binocular autorefractor/keratometer was used to objectively evaluate her refractive response and pupil reaction according to accommodative stimulation. The IOL Master was used to evaluate the anterior chamber depth (ACD), lens thickness (LT), and pupil diameter with relaxed and strained eyes. For stepwise static accommodative stimuli (1–5 D), the refractive responses were correspondingly stepwise, similar to those elicited by healthy individuals. However, contrary to physiological accommodation, she strained her eyes to see distant objects and relaxed them to see near objects. There was no change in pupil diameter despite the accommodative stimuli being maximum. Biometry results showed that ACD deepened and LT flattened with eye strain, which were contrary to those during physiological accommodation.

**Conclusions:**

We report a rare case with reverse of physiological accommodative response. When patients complain of unusual distant visual discomfort, pseudomyopia with paradoxical accommodation should be considered.

## Background

Accommodation involves changing the optical power to sharply focus on objects placed at varying distances. Although accommodation can be consciously controlled, it usually acts as a reflex. In response to accommodation stimuli, the ciliary muscles contract to thicken the lens, and convergence and miosis occur [1]. A complex network of parasympathetic nervous system controls this reflex through the Edinger-Westphal nucleus.

Spasm of the near reflex involves excessive accommodation, excessive convergence, and miosis with varying severity, combinations, and duration. Excessive accommodation, pseudomyopia, can occur separately, and result in blurred distance vision due to increased refractive power generated by ciliary muscle spasm, asthenopia, and headache. Pseudomyopia may develop because of organic reasons, parasympathetic nervous system stimulation, or functional reasons, such as eye strain, long-term near work, or emotional stress [2–6]. Multidisciplinary treatments, including strong cycloplegics for relaxing ciliary muscle spasm, psychological consultation, and modification of near work environment, are needed for patients with pseudomyopia because of their inability to voluntarily relax the ciliary muscle spasm [7].

Herein, we report the first case of a patient with paradoxical accommodation who could reverse pseudomyopia by straining the eyes, which was contrary to physiological accommodation.

## Case presentation

A 33-year-old woman without any history of trauma presented with a 3-year-history of poor distant vision. She could not see distant objects sharply and felt as if a layer of vinyl covered her eyes. However, she could see near objects clearly. This occurred at least a few hundred times daily. She could see distant objects sharply by straining her eyes. She could voluntarily strain her eyes to see clearly at distance and actually, she constantly has been straining her eyes to see clearly at distance. Her uncorrected visual acuity was 20/20 during staining, and 20/50 during relaxing. These ocular symptoms began after she experienced tremendous stress because of her frequent migration abroad.

Her uncorrected distance and near vision were 20/20. During manifest refraction (MR), an unusual reaction was observed. MR in both eyes in the relaxed state was approximately − 2.5 diopter (D). While focusing on distant letters, she grimaced and strained her eyes, following which, her MR changed to approximately − 0.5 D. These movements were constantly repeated throughout the MR assessment. Her response was contrary to the physiological accommodative response. The cover–uncover test for ocular alignment showed orthotropia at far and near and orthophoria at distance regardless of eye straining or relaxing. To exclude neurological abnormalities, she had tests including direct and indirect light reflex, visual field test using a Humphrey visual field analyzer, Ishihara color perception test, and visual evoked potential test. All test results were within normal limit. Basic blood test showed no abnormalities, however, brain imaging test was not performed due to cost.

Her refractive response and pupil change to accommodative stimulations were objectively evaluated with a WAM-5500 binocular autorefractor/keratometer (Grand Seiko Co. Ltd., Hiroshima, Japan). This instrument gives a binocular open view through a transparent acrylate panel, providing an unrestricted view of objects at all distances. The refractive and pupillary changes while gazing at a far object with relaxed (far relax) and strained (far strain) eye states, and at a near object (33 cm) with relaxed eye state (near relax), were measured. Next, we recorded refractive and pupillary changes at static stimulation and dynamic stimulation. Static stimulation was performed at five accommodative stimuli; 1 D (100 cm), 2 D (50 cm), 3 D (33 cm), 4 D (25 cm), and 5 D (20 cm). Dynamic stimulation was performed with a moving target at a speed of 300 mm/s from 100 to 33 cm. Two sets of measurements were performed on the same day. One set comprised 10 measurements, with sufficient rest between the examinations. The same examination was performed after 1 week. Finally, the mean value of 40 measurements with the standard deviation was plotted. The IOL Master 700 (Carl Zeiss Meditec AG, Jena, Germany) was used to evaluate the anterior chamber depth (ACD), lens thickness (LT), and pupil diameter with relaxed and strained eyes. Measurements were performed in four sets (one set of three measurements).

**Fig. 1:**
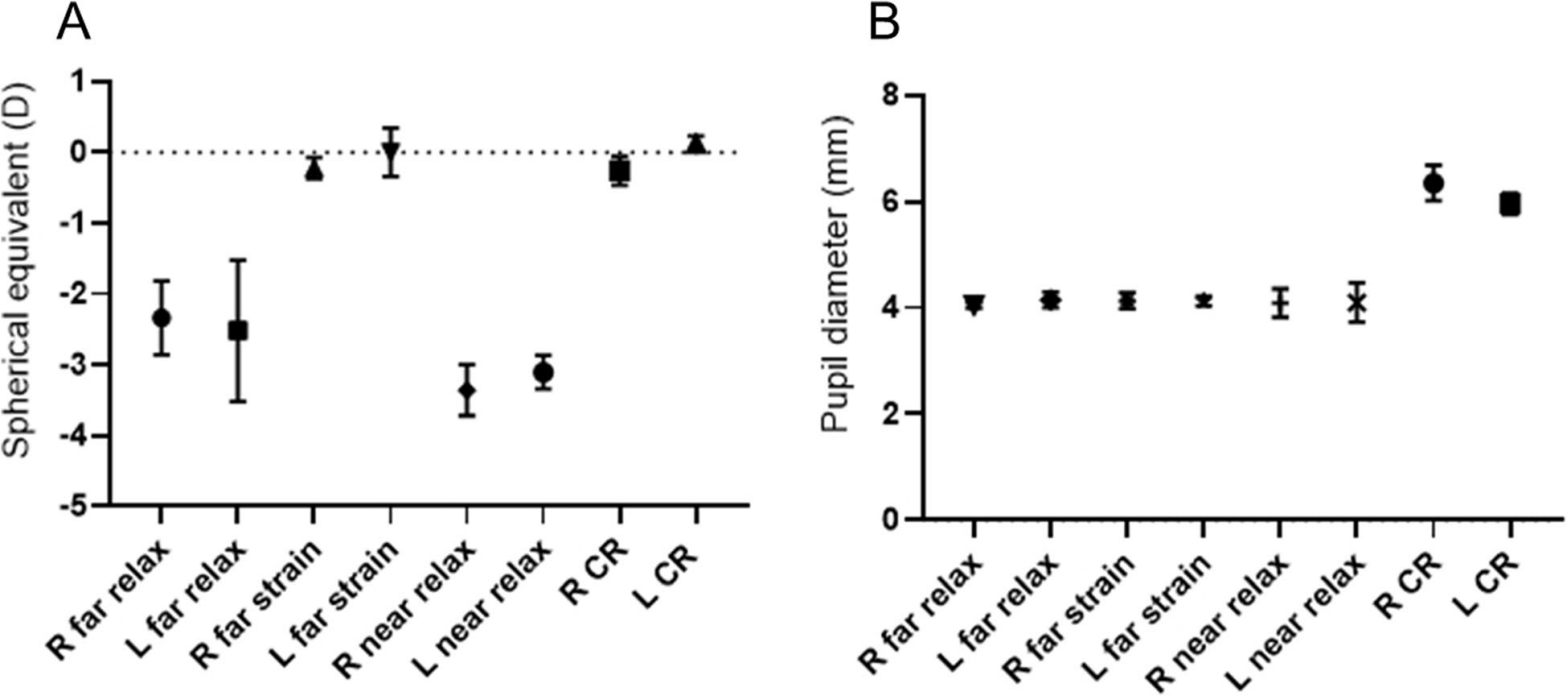
**a** Manifest refraction of both eyes at far distance is approximately − 2.5 D myopia under relaxed eye state (far relax) and emmetropia under strained eye state (far strain). Both eyes have approximately − 3.0 D myopia in the relaxed state at near distance (33 cm; near relax) and emmetropia under cycloplegic refraction (CR). **b** Pupil diameter shows no significant change according to the patient’s eye state, except during the CR state (Kruskal-Wallis test; *P* = .59). L = left; R = right

In the far relaxed state, the spherical equivalent (SE) was − 2.34 D in the right eye and − 2.50 D in the left eye; however, in the far strained state, for a sharp and clear distance vision, SE was − 0.22 D and 0.00 D in the right eye and left eye, respectively. In the near relaxed state, SE in the right eye and left eye was − 3.36 D and − 3.10 D, respectively. Cycloplegic refraction (CR) in the right eye and left eye was − 0.26 D and 0.13 D, respectively (Fig. 1a). Her pupil diameter was approximately 4 mm, without any significant difference in size while gazing at a distant or near object (Kruskal-Wallis test, *P* = .59) (Fig. 1b).

**Fig. 2:**
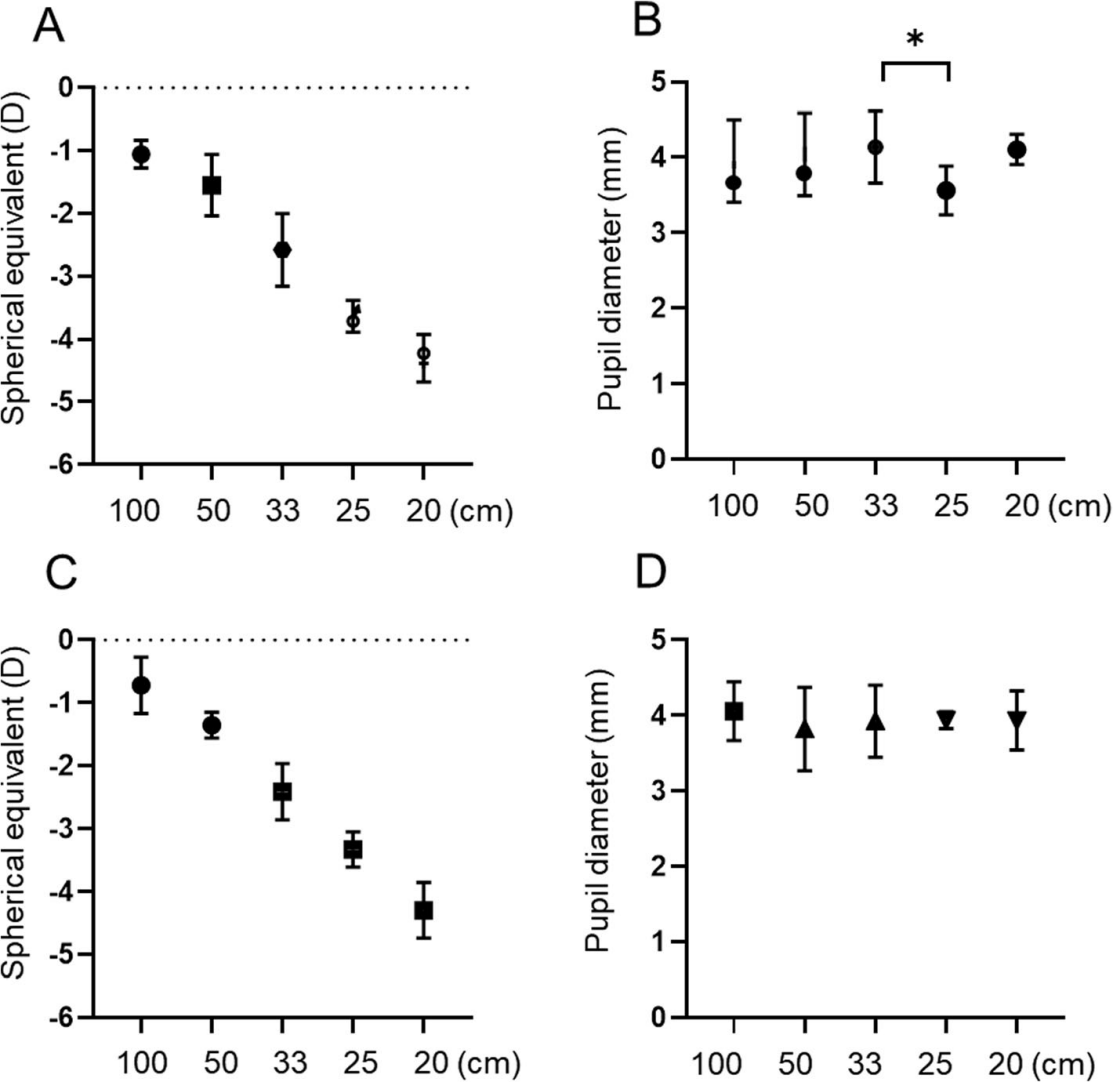
Changes in the accommodative response and pupil diameter according to static refractive stimuli (100, 50, 33, 25, and 20 cm). **a**, **b** Accommodative response of the right eye shows significant changes according to refractive stimuli. However, there is no significant change in pupil diameter according to the refractive stimuli, except between 33 and 25 cm (Kruskal-Wallis test, Dunn’s multiple comparison test, *P* = .005). **c**, **d **Accommodative response of the left eye shows significant changes according to the refractive stimuli; however, there is no significant change in pupil diameter according to the refractive stimuli. *P-value by Kruskal-Wallis test, Dunn’s multiple comparison test; significance was set at < 0.05

**Fig. 3:**
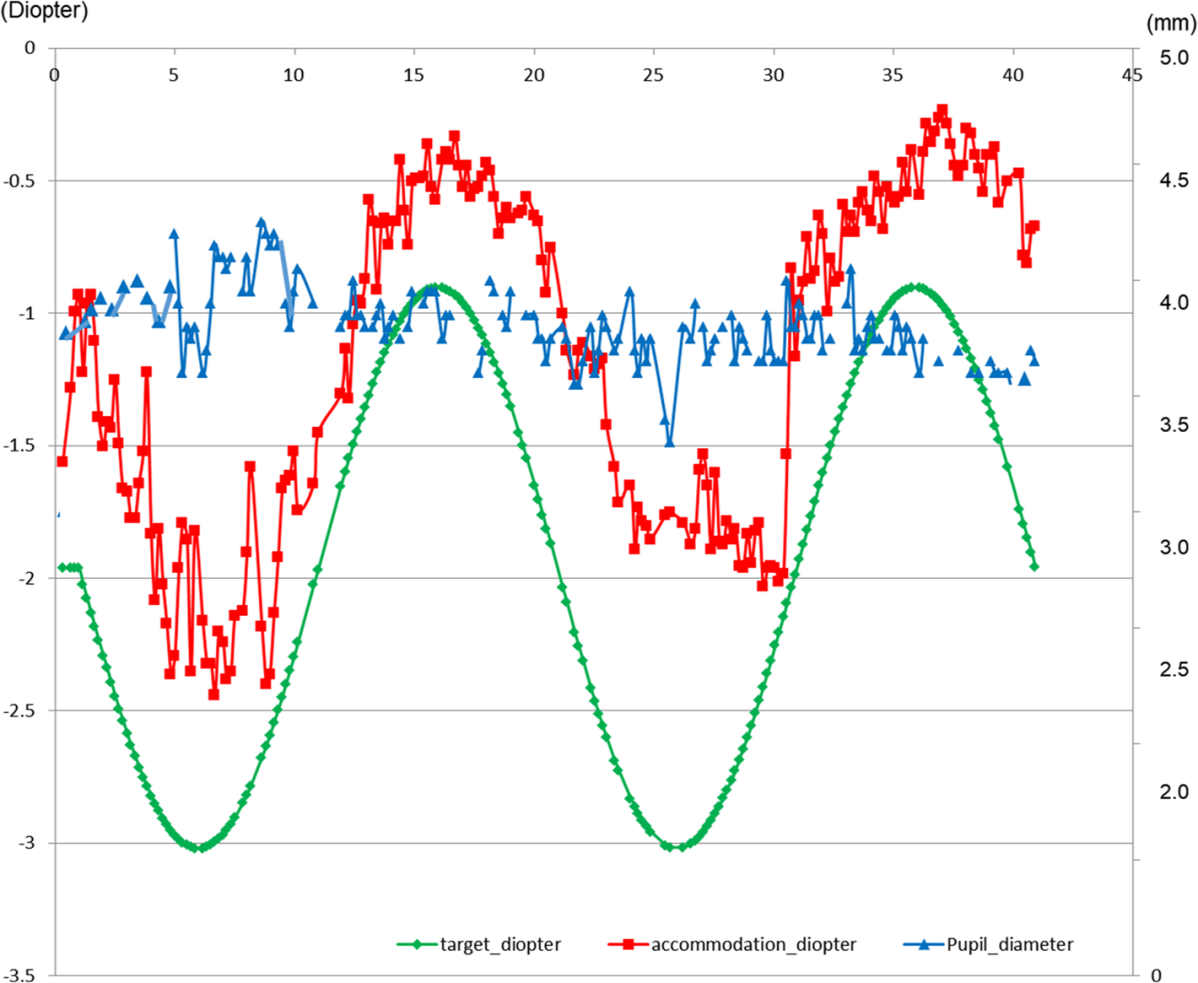
Dynamic measurement of refractive responses according to accommodative stimuli from 100 to 33 cm (from − 1 D to − 3 D). The patient’s refractive responses demonstrated by the red curve smoothly followed the green target stimulus, with a refractive power of about 70 % of the actual stimulus. However, pupil diameter did not show any change according to accommodative stimulus

For strong static accommodative stimuli (1–5 D), the refractive responses were proportionately strong, similar to those elicited by healthy individuals (Fig. 2a, c) [8]. Contrary to physiological accommodation, she strained her eyes to see distant objects and relaxed her eyes to see near objects. Pupil diameters showed no difference despite strong accommodative stimuli, except between 33 and 25 cm in the RE (Kruskal-Wallis test, Dunn’s multiple comparison test; *P* = .005) (Fig. 2b and d). In the dynamic stimulation test, she could follow the moving target from 100 to 33 cm without any constraint, and her refractive response was similar to that during normal reaction. Similarly to that observed during the static test, her pupil diameter showed no change during the dynamic test (Fig. 3).

Biometry results indicated that ACD deepened and LT flattened with maximal eye strain, compared with relaxed eyes, and her pupil diameter showed no significant change (Table 1). These results were contrary to the physiological movements of ACD and LT.

**Table 1:** Changes in the anterior chamber depth, lens thickness, and pupil diameter measured using the IOL Master under relaxed and strained eye states

	Right eye			Left eye		
	Relaxed	Strained	*P*-value*	Relaxed	Strained	*P*-value*
Anterior chamber depth (mm)	3.59±0.01	3.63±0.05	0.17	3.58±0.02	3.66±0.05	0.03
Lens thickness(mm)	3.64±0.00	3.62±0.01	0.08	3.66±0.01	3.62±0.00	0.04
Pupil diameter (mm)	3.97±0.56	3.74±0.44	0.12	4.15±0.94	4.11±0.74	0.69

*P-value by Wilcoxon signed rank test; significance is set at *P* <.05

We diagnosed these rare reverse eye responses to accommodation as paradoxical accommodation. She has provided informed consent for publication of the case.

## Discussion and conclusions

We report the case of a patient who, despite poor distant vision (− 2.5 D myopia) during relaxed eye state, showed paradoxical accommodation and manifested emmetropia by straining her eyes to see distant objects. She showed normal refractive responses to various accommodative stimuli; however, the tension in her eyes was contrary to that manifested during normal physiological reaction (emmetropia with strained eyes; myopia with relaxed eyes). Pupil diameter remained unchanged despite increased accommodative stimulus. On biometry, her ACD deepened and LT flattened with strained eyes and vice versa with relaxed eyes.

MR was the most important clue for establishing a definite diagnosis. Her refraction changed continuously, minute by minute. She had to strain her eyes and grimace to see distant objects. She was eventually diagnosed with pseudomyopia due to poor distant vision, with concomitant myopia, good near vision, and emmetropia in CR. Psychological stress may evoke parasympathetic spasm, and she might have adapted to consciously control her pseudomyopia. This was confirmed by the presence of an unstable refractive change with a large standard deviation of MR while consciously controlling her eyes to look at distant objects. In contrast, a stable myopic state was maintained with a small standard deviation of MR while looking at near objects with relaxed eyes (Fig. 1a).

Although the patient’s refractive responses for accommodative stimuli were the same as those of a healthy individual, the straining and relaxation of her eyes were opposite to those manifested during physiological accommodation. Pupil diameter showed little change during static and dynamic accommodative stimulations, and thus, we assumed that pupillary reactions were paradoxical. The pupil size reduced as the accommodative stimuli became stronger during physiological accommodation, because the pupils constrict to increase the depth of focus while looking at nearby object sharply; this response is mediated by the parasympathetic nervous system [8, 9]. No response of the pupil suggests that parasympathetic-iris connection was faulty.

During dynamic stimulation, our patient’s refractive responses followed the physiological accommodative stimulus with a refractive power of approximately 70 % of the actual stimulus (Fig. 3). Generally, the actual refractive response and pseudoaccommodation, including changes in pupil diameter, ACD, and corneal aberrations, account for 90.9 and 9.1 % of the normal refractive response to the accommodative stimulus in healthy subjects, respectively [8]. The patient’s relatively insufficient refractive response to the accommodative stimulus was not due to insufficient refractive power but inadequate time to exert the accommodative response because the target moved too fast to be thoroughly followed. This phenomenon appears to be the same in healthy individuals. Therefore, Fig. 3 should not be interpreted as insufficient power of refractive response by the patient.

We suggest three possible hypotheses for this abnormal reaction. First, the patient consciously adapted to overcome pseudomyopia by straining her eyes, similar to that when viewing an autostereogram (a two-dimensional image) that induces a visual illusion of a three-dimensional image. Depth perception requires training to overcome the normal coordination between accommodation and convergence, with disruption of the near reflex [10]. Our patient learned to relax the ciliary spasm by eye straining, thereby reversing physiological accommodation. Second, an unknown organic problem decoupled the physiological parasympathetic-ocular circuit. Distorted parasympathetic nervous innervations to the eyes resulted in unusual ocular responses to parasympathetic stimulation, similar to the Marcus-Gunn jaw-winking phenomenon [11]. The fact that her pupil size remained unchanged during accommodation supports this hypothesis. Third, she learned to exert sympathetic stimulation to inhibit the parasympathetic spasm, possibly by an autonomic dysfunction, although voluntary control of the autonomic nervous system is considered impossible.

Treatment of pseudomyopia depends on the underlying etiology. Correction of organic causes, including systemic disease [12], systemic or ocular medications [5, 13], head injury [3, 4] and brain disease [14], or uveitis [15], would be a useful strategy. Functional stimulation of the parasympathetic tone can be corrected by cycloplegics, psychological support, modification of working conditions, and appropriate ocular exercise and relaxation. We prescribed atropine to the patient to relieve eye strain during distant vision; however, she refused it because of poor near vision. Therefore, we prescribed − 1.0-D glasses to minimize asthenopia induced by continuous eye strain; she was satisfied with these glasses. We could not figure out the exact cause of her phenomenon. Currently, she has normal ciliary muscle power, therefore, she overcome her accommodative spasm at distance. However, if she got older and had presbyopia, she could have an unexpected reaction.

In conclusions, most patients with pseudomyopia cannot overcome the condition by themselves. However, some patients can consciously overcome pseudomyopia through paradoxical accommodation, by straining their eyes to induce emmetropia when looking at distant objects, and relaxing their eyes when focusing at near objects. When patients complain of unusual distant visual discomfort, pseudomyopia with paradoxical accommodation should be considered with detailed history taking and MR examination.

## Data Availability

All date generated and analyzed during this study are included in this article.

## Abbreviations

A: Accommodation
ACD: Anterior chamber depth
CR: Cycloplegic refraction
D: Diopter
LT: Lens thickness
MR: Manifest refraction
SE: Spherical equivalent

## References

[1] Roy FH (2001). Mechanism of Accommodation in Primates. Ophthalmology.

[2] Cogan DG, Freese CG (1955). Spasm of the Near Reflex. AMA Arch Ophthalmol.

[3] Hughes FE, Treacy MP, Duignan ES, Mullaney PB (2017). Persistent Pseudomyopia Following a Whiplash Injury in a Previously Emmetropic Woman. Am J Ophthalmol Case Rep.

[4] London R, Wick B, Kirschen D (2003). Post-traumatic Pseudomyopia. Optometry.

[5] Stratos AA, Peponis VG, Portaliou DM (2011). Secondary Pseudomyopia Induced by Amisulpride. Optom Vis Sci.

[6] Keane JR (1982). Neuro-ophthalmic Signs and Symptoms of Hysteria. Neurology.

[7] Jones R (1990). Physiological Pseudomyopia. Optom Vis Sci.

[8] Park H, Park IK, Shin JH, Chun YS (2019). Objective Verification of Physiologic Changes during Accommodation under Binocular, Monocular, and Pinhole Conditions. J Korean Med Sci.

[9] Wang B, Ciuffreda KJ (2006). Depth-of-focus of the Human Eye: Theory and Clinical Implications. Surv Ophthalmol.

[10] Yankelevsky Y, Shvartz I, Avraham T, Bruckstein AM (2016). Depth Perception in Autostereograms: 1/f Noise is Best. J Opt Soc Am A Opt Image Sci Vis.

[11] Gunn RM. Congenital Ptosis with Peculiar Associated Movements of the Affected Lid. Trans Ophthal Soc UK, 1883;3:283–7.

[12] Romano PE, Stark WJ (1973). Pseudomyopia as a Presenting Sign in Ocular Myasthenia Gravis. Am J Ophthalmol.

[13] Padhy D, Rao A. Bimatoprost (0.03 %)-Induced Accommodative Spasm and Pseudomyopia. BMJ Case Rep 2015;2015. 10.1136/bcr-2015-211820. 10.1136/bcr-2015-211820 PMC4680562 26598527

[14] Voon LW, Goh KY, Lim TH, Tan KK, Yong VS (1997). Pseudomyopia in a Patient with Blocked Ventriculo-peritoneal Shunt–a Case Report. Ann Acad Med Singapore.

[15] Ijaz U, Habib A, Rathore HS (2018). A Rare Presentation of Cyclitis Induced Myopia. J Coll Physicians Surg Pak.

